# Electrocatalytic Oxidation of Methanol by a Polymeric Ni Complex-Modified Electrode Prepared by a One-Step Cold-Plasma Process

**DOI:** 10.3389/fchem.2020.595616

**Published:** 2020-12-10

**Authors:** Ji-Soo Jeon, In-Keun Yu, Woonjung Kim, Seong-Ho Choi

**Affiliations:** ^1^Department of Chemistry, Hannam University, Daejeon, South Korea; ^2^Plasma Technology Research Center, National Fusion Research Institute, Gunsan, South Korea

**Keywords:** one-step cold-plasma process, cyclic voltammetry, electrocatalytic oxidation, Ni-modified catalytic electrode, methanol, polymeric nickel complexes

## Abstract

In this work, a polymeric nickel complex-modified indium tin oxide (ITO) electrode was prepared by a one-step cold-plasma process of acrylic-Ni complex precursors. Also, the work provides the electrocatalytic oxidation of methanol by a polymeric Ni complex-modified electrode prepared by a simple one-step cold-plasma process. The acrylic-Ni complex precursors were synthesized by complexation of nickel (II) chloride, and acrylic acid in a small amount of water; subsequently we added N,N′-methylene-bis-acrylamide as a crosslinking agent to the complex solution. We characterized the prepared polymeric Ni complex-modified (Ni-modified) catalytic electrode by X-ray photoelectron spectroscopy, field emission scanning electron microscopy, and electrochemical methods. Electrochemical characterization showed stable redox behavior of Ni(III)/Ni(II) couples. Cyclic voltammetric experiments have shown that electrocatalytic oxidation of methanol can occur on Ni-modified catalytic electrodes, while not observed on bare ITO. As a result, this work provides the simple and easy preparation of electrocatalysts by one-step plasma process for methanol fuel cell.

## Introduction

Electrocatalytic oxidation of methanol is an intensive research topic on direct methanol fuel cells (DMFC) as power sources for electric vehicles and electronic devices (Shukla et al., 1998; Scott et al., 1999; Kamarudin et al., 2009; Achmad et al., 2011). Recently, considerable efforts have been focused on the study of electrocatalytic oxidation in alkaline solutions (Taraszewska and Roslonek, 1994; Yi et al., 2007; Danaee et al., 2008; Rosca and Koper, 2008; Ojani et al., 2009a). The application of alkaline solution to a fuel cell has many advantages, such as increased efficiency (Wang et al., 2015) and providing a wider selection of possible electrode materials and better efficiency of the oxygen cathode. The oxidation reaction of organic fuels is almost insensitive to the surface structure. Smaller or negligible poisoning effects in alkaline solutions are observed. In this context, electrode materials are clearly important parameters in the electrochemical oxidation of methanol, where highly efficient electrocatalysts are needed. Nickel as a low cost, relatively abundant material has demonstrated long-term stability in alkaline solutions (Jafarian et al., 2006; Spinner and Mustain, 2011; Wang et al., 2011); hence it is a useful catalyst for methanol oxidation.

Nickel oxide is widely studied because it can be applied to fuel cell electrodes, electrochromic films, electrochemical capacitors, optical materials, batteries, photocatalysts, etc. (Hu and Cheng, 2002; Wu et al., 2009, 2010). Most of these useful features mostly depend on the composition, morphology, and structure of the nickel oxide deposits. It is believed that the nanostructured nickel oxide has better properties than the bulk counterparts (Sk et al., 2016). There are many different methods reported for synthesis of the nanostructured nickel oxides (Ghanem et al., 2015; Razali et al., 2017). Some of these methods for making nickel oxide powder have difficulties in the manufacture of well dispersed nickel oxide electrodes for electrochemical applications. The nickel-modified electrodes were Ni/polymers (Ojani et al., 2008, 2009a,b; Raoof et al., 2010, 2011). The prepared Ni-modified electrodes can successfully catalyze the methanol oxidation in an alkaline medium (Ojani et al., 2011). In our experience, these methods do not work well, because the homogeneous slurry could not be made by mixing the Ni power and polymer as binders in a fuel-cell electrode. On the other hand, Taraszewska and Roslonek (1994) found that glassy carbon/Ni(OH)_2_ modified electrode acts as an effective catalyst for the oxidation of methanol.

On the other hand, the cold-plasma processes have recently received much interest for growing polymer films on different substrates (Wang et al., 2018). O'Hare et al. (2006) could obtain the organic material coating by the deposition of acrylic-acid (AA) monomer at a deposition rate of 40 nm min^−1^ in post discharge of a radio-frequency atmospheric-pressure helium plasma torch. Nisol et al. (2013) polymeized the AA film by spraying the precursor to helium plasma using a dielectric barrier discharge process. Depending on the helium and organic precursor flow rates, fragmentation of AA monomers can be controlled to obtain different concentrations of carbonyl groups in the coating (Beck et al., 2008). AA coatings were very sensitive to the deposition parameters, which could affect the film functionalization and the stability in water. There is no report as yet of preparation of Ni-modified electrodes by a one-step cold-plasma process. In this paper, we first prepared the Ni-modified (polymeric Ni complex) catalytic electrodes by a one-step cold-plasma process, and investigate the electrochemical oxidation of methanol to direct methanol fuel-cell catalysts (Mahapatra et al., 2011). We found that Ni-modified electrodes can electro-oxidize the methanol with high current densities.

## Materials and Methods

### Reagents

We purchased:

- Sodium phosphate monobasic dihydrate (NaH_2_PO_4_.2H_2_O), N,N′-Methylenebisacrylamide, ascorbic acid, sodium hydroxide, sodium chloride, uric acid, copper (II) chloride dihydrate, iron (III) chloride hexahydrate, zinc (II) chloride dehydrate, and sodium phosphate dibasic dihydrate (Na_2_HPO_4_.2H_2_O) from Sigma-Aldrich.
- Potassium ferricyanide (K_3_Fe(CN)_6_), potassium chloride, potassium ferrocyanide (K_4_Fe(CN)_6_.3H_2_O), hydrogen peroxide (H_2_O_2_, 30%) from Duksan Pharmaceutical Co., Ltd. (Korea).
- Acrylic acid and hydrochloric acid from Alfa Aesar.
- ITO coated glass as working electrode (30–60 Ω/sq, 25 × 25 × 1.1 mm) from Sigma-Aldrich.

A phosphate buffer was prepared by mixing 0.1 M NaH_2_PO_4_ and 0.1 M Na_2_HPO_4_, and then the pH was adjusted to a value of 7.4. We have prepared a water purification experiment solution by Milli Q plus water purification system (Millipore, Co., Ltd.; final resistance of water is 18.2 MΩ cm^−1^).

### Instruments

We characterized the surface properties with contact angle measuring device (PHOENIX 300, Surface Electro Optics Co., Ltd., Korea), scanning electron microscopy (FE-SEM (S 4800), Hitachi, Tokyo, Japan) and X-ray photoelectron spectroscopy (MultiLab. ESCA 2000, Thermo Fisher Scientific, Inc., USA). We performed cyclic voltammetry (CV) using a VersaSTAT 3 potentiostat/galvanostat (AMETEK PAR, USA) and a conventional three-electrode system consisting of ITO glass as the working electrode, a platinum wire as the counter electrode, and Ag/AgCl as the reference electrode.

Plasma devices had to be small, simple, and portable for a variety of applications. We have chosen a cold-plasma device that is closest to these requirements, the power that can be controlled from 200 to 250 W. Most of the experiments were conducted in the 200 W range. The torch used in this experiment is conical and the diameter of the torch tip is 2.0 mm. Nitrogen was supplied as the working gas from a tank connected to a plasma generator with an internal flow controller. Plasma generators were used to maintain the pressure at a constant level. The stock solution was injected into the cold-plasma jet by a syringe pump in the downstream region. The ITO substrate can be placed under a cold-plasma jet at a distance of 1 cm and moved manually during the deposition process.

### Fabrication of Ni-Modified Catalytic Electrode

We prepared the precursor solutions for the experiment as follows. First, an exact amount of nickel chloride salt was dissolved in 0.5 ml of purified water, and then mixed with 5 ml of acrylic acid, and then N,N′-Methylenebisacrylamide of 0.5 g was added the prepared mixture solution. The ITO substrate was pre-washed using ethanol and ultrasonicated for 10 min before cold-plasma deposition. In the course of the deposition process, the ITO substrate was slowly moved in the x and y directions, and the deposition time was 5 min. The precursor solution was injected into the cold-plasma jet using a syringe pump at a flow rate of 0.3 mL/min, and the gas pressure was kept constant at 0.018 MPa. After the deposition process was completed, the prepared electrode was washed with methanol and water to remove unreacted precursors, then dried and stored at 4°C before application.

## Results

### Contact Angle Analysis

**Figure 1:**
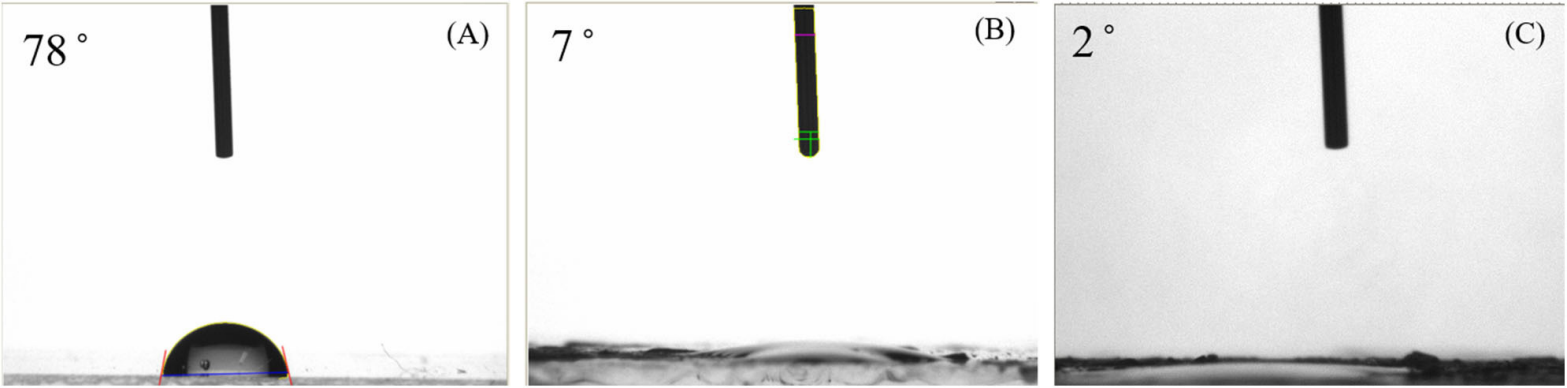
Contact angles of the bare ITO glass surface **(A)**, ITO glass surface irradiated by plasma **(B)**, and Ni-modified catalytic electrode **(C)**.

Figure 1 shows the contact angles of the fabricated Ni-modified catalytic electrode by cold-plasma deposition. From an examination of the water-contact angles, we found that the contact angle of the bare ITO electrode (A) and of the plasma-irradiated ITO electrode (B) was 78° and below 10.0°, respectively. The contatact angle of the Ni-modified catalytic electrode (C) was <5.0°, as shown in the results in Figure 1. As can be seen from results, the original ITO glass is almost hydrophobic, with a high contact angle compared to that of the irradiated ITO glass surface, which was introduced hydroxy group (-OH) during the cold-plasma process. Compared to original ITO glass, the contact angles of the fabricated Ni-modified catalytic electrode were significantly lower, because there is a large amount of functional carboxyl group (-COOH) from poly(acrylic acid), PAA, which has strong hydrophilic properties. The presence of a hydrophillic metallic hydroxide on each electrode also transfers hydrophobic ITO to hydrophillic properties. From these results, we confirm that the surface modified by the metallic hydoxide/PAA composites over ITO electrodes has hydrophillic properties.

### Scanning Electron Microscopy (SEM) Analysis

**Figure 2:**
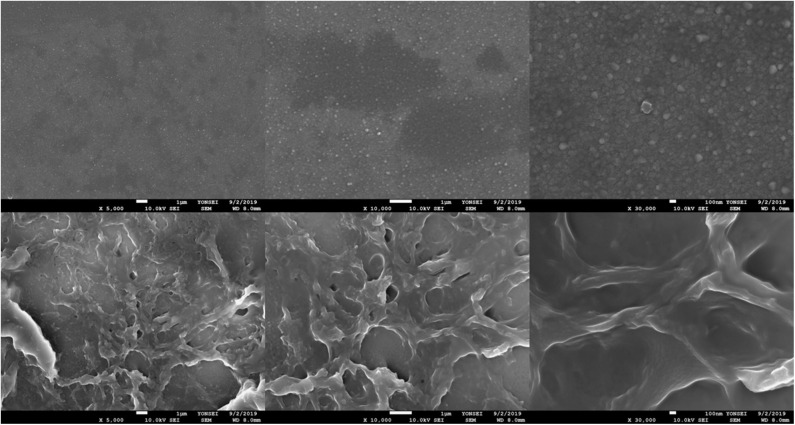
SEM images of the bare ITO glass surface (**upper**) and the Ni-modified catalytic electrode (**bottom**).

We characterized the surface morphologies of the bare ITO electrode (upper) and the Ni-modified catalytic electrodes (bottom) by scanning electron microscopy (SEM) (Figure 2). The Ni-modified catalytic electrode surface shows a net-like morphology suggesting the successful deposition of Ni/PAA onto the ITO substrate.

### Energy-Dispersive X-Ray Spectrometry (EDS) Image Analysis

**Figure 3:**
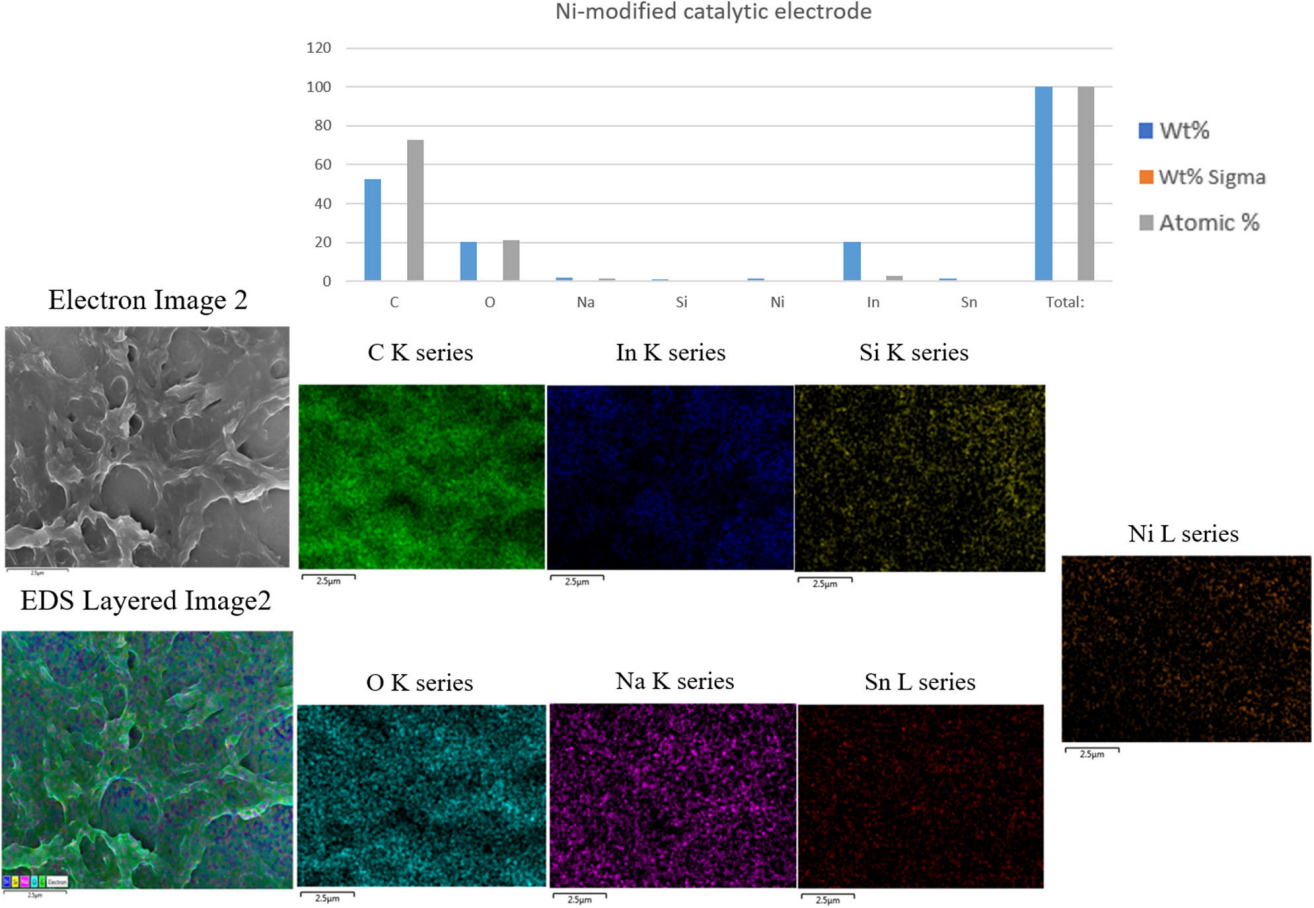
SEM-EDS Layered images of the Ni-modified catalytic electrode prepared by one-step cold plasma processing.

Figure 3 shows SEM-EDS layered images of the Ni-modified catalytic electrode prepared by one-step cold-plasma processing. As can be seen, the EDS images of the fabricated Ni-modified catalytic electrode show significant spots of carbon, oxygen, and Ni on their surfaces. This result suggested the successful depositing of Ni/PAA on the ITO electrodes by cold-plasma deposition.

### Energy-Disperisve X-Ray Spectrometry (EDS) Analysis

**Figure 4:**
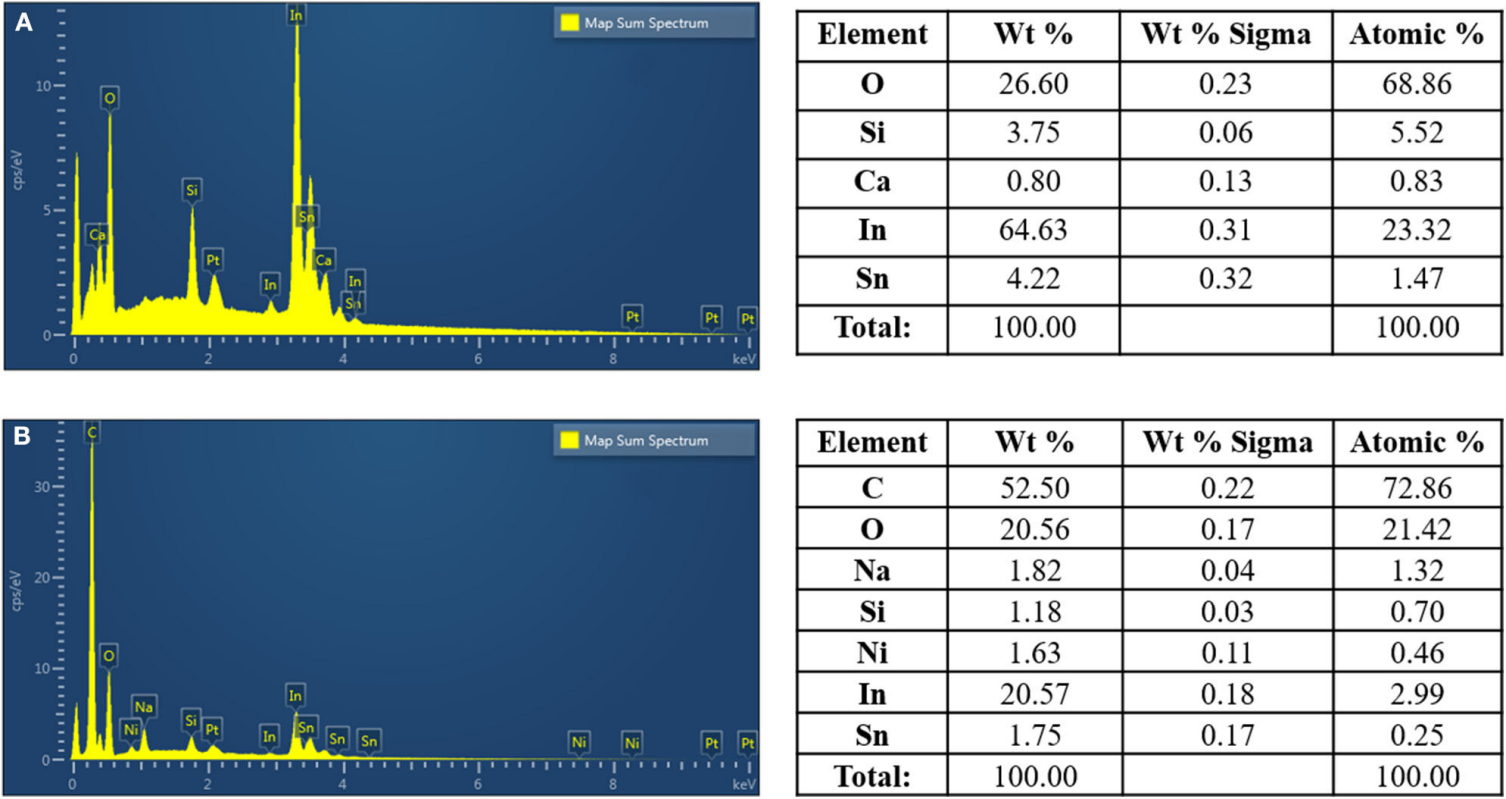
SEM-EDS imaged of ITO glass surface **(A)**, and the Ni-modified catalytic electrode **(B)**.

Figure 4 exhibits the EDS data of the Ni-modified catalytic electrodes prepared by cold-plasma deposition. As can be seen, the EDS spectra of the fabricated Ni-modified catalytic electrode show a significant increase in the amount of carbon, oxygen, and Ni on their surfaces, when compared to the bare ITO electrode. This results suggests the successful depositing of Ni/PAA on the ITO electrodes by cold-plasma deposition.

### X-Ray Photoelectron Spectroscopy (XPS) Analysis

**Figure 5:**
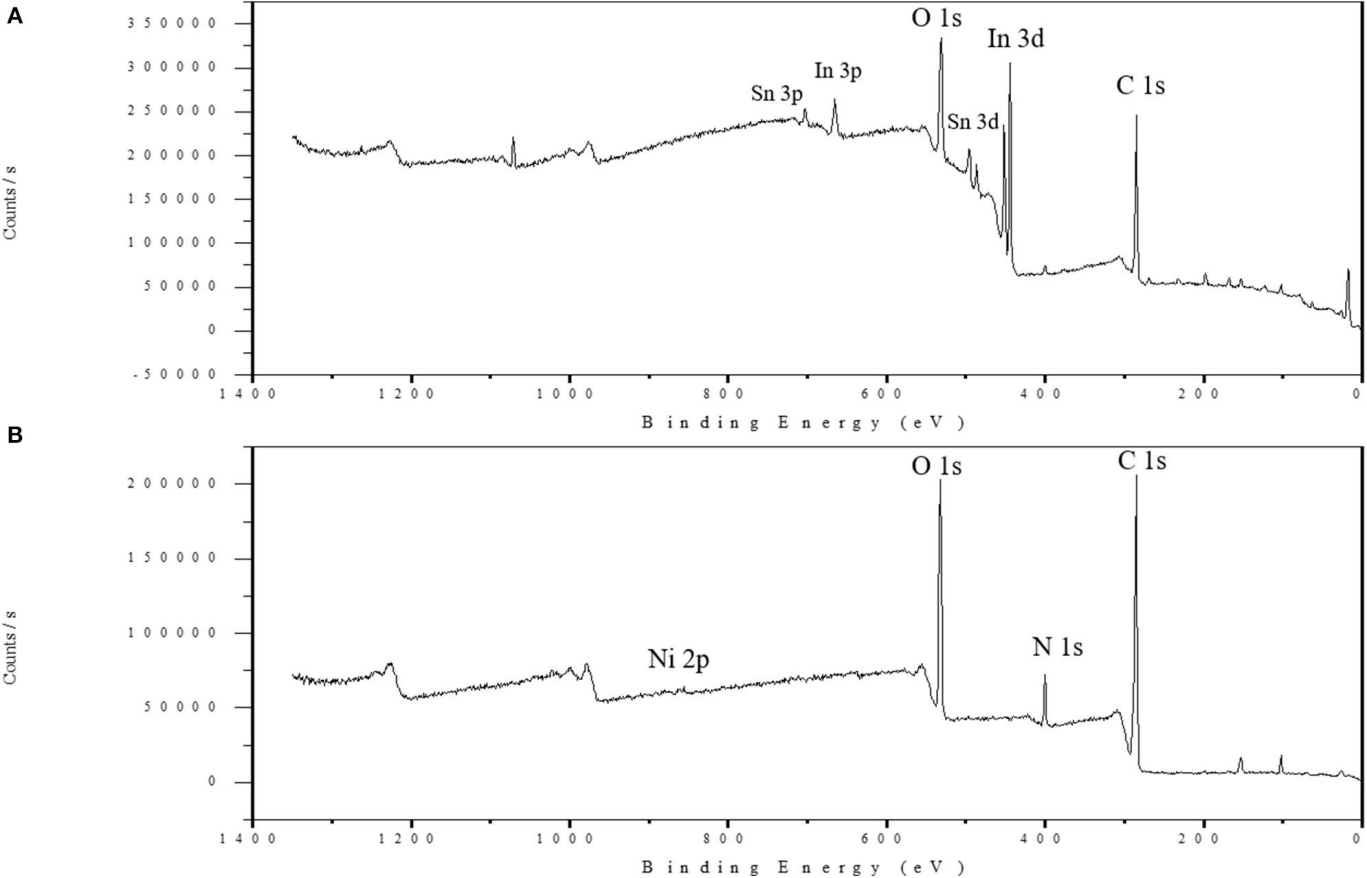
XPS survey scan spectra of the bare ITO glass surface **(A)** and the Ni-modified catalytic electrode **(B)**.

Figure 5 displays the XPS survey scan spectra of the Ni-modified catalytic electrodes fabricated by a cold-plasma process. In the bare ITO electrode surface of Figure 5A the In and Sn peaks could be observed, but the In and Sn peaks have disappered after the polymer was depositedonto the surface of the ITO glass. In Figure 5B, the two peaks at 853.3 and 834.9 eV, belonging to the binding energies of NiO 2p3/2 and NiO 2p1/2, indicate the presence of Ni in the electrode. Additionally, the peaks at 400 eV suggest that the Ni is in the form of cross-linked chemicals. On the other hand, the O 1s peak appears around 531.8 eV, which indicates the presence of a carboxylic acid of PAA in the electrode.

### Cyclic Voltammetry Analysis

**Figure 6:**
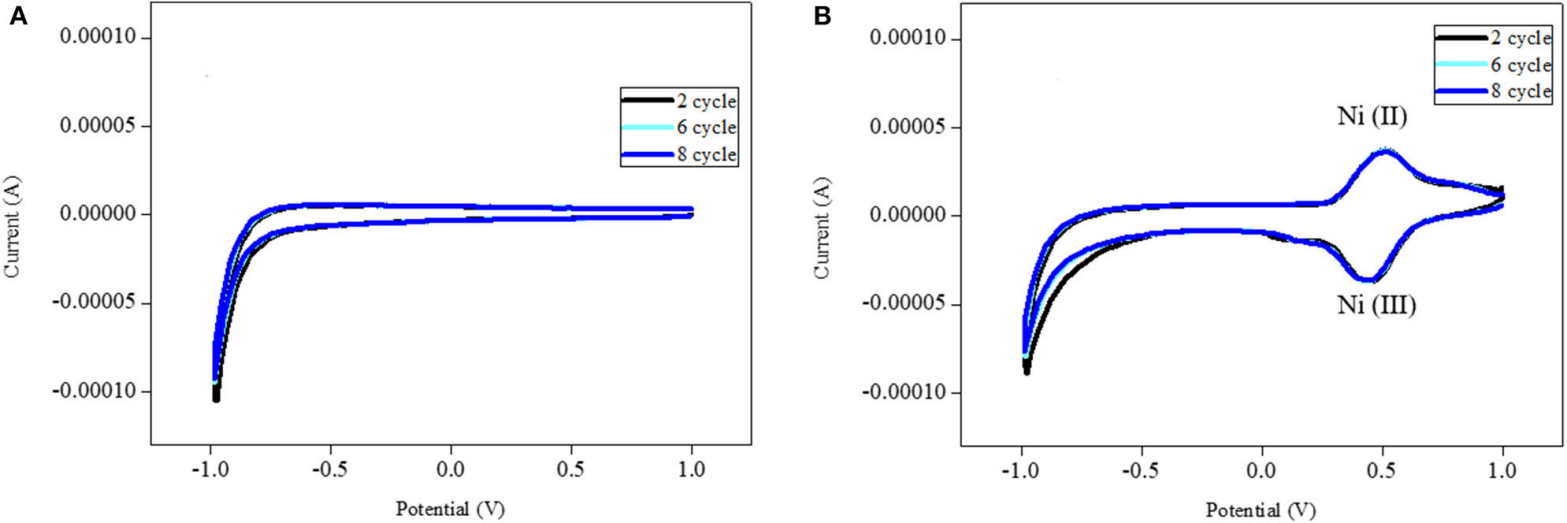
Cyclic voltammograms of the base ITO electrode **(A)** and Ni-modified catalytic electrode **(B)** in 0.1 M PBS electrode with scan rate of 100 mV/min.

We used cyclic voltammetry to study the electrochemical performance of the fabricated Ni-modified catalytic electrodes. Figure 6 shows the cyclic voltammetry (CV) of these electrodes in 0.1 M phosphate buffer solution of pH = 7.4. The bare ITO electrodes did not show any electrochemical redox peaks. However, the Ni-modified catalytic electrode showed predominate redox peaks that correspond to the respective metal complexes with high currents. We noticed that the electrochemical stablity of the Ni-modified catalytic electrode was low, because they show deteriorating current densities over the continuous potential cycles (Bui et al., 2019). In order to solve this problem, we added N,N′-methylenbis(acrylamide) (MBA) as the crosslinking agent to the precursor solution. As we expected, the stability of the Ni-modified catalytic electrode increased significantly during cyclic voltammetry performance, which makes it such a good candidate for MeOH oxidation.

### Electrocatalytic Evaluation of Ni-Modified Catalytic Electrode to MeOH

**Figure 7:**
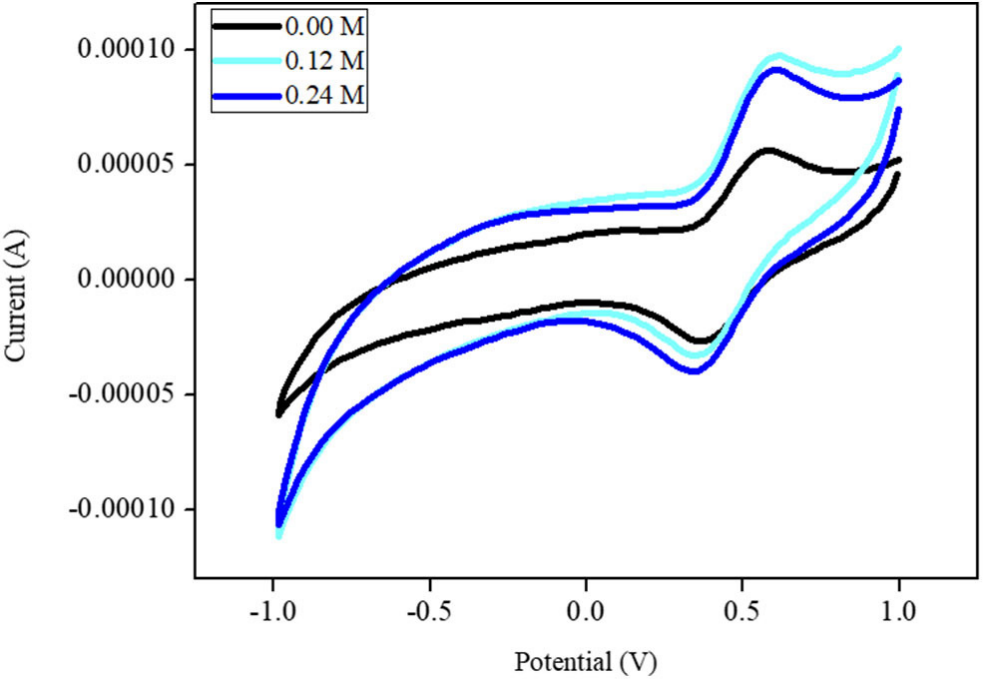
Cyclic voltammograms of MeOH oxidation using Ni-modified catalytic electrode in 0.1 M PBS with scan rate of 100 mV/min.

Figure 7 shows the CVs of the Ni-modified catalytic electrodes for MeOH oxidation. The CV of the Ni-modified catalytic electrodes shows the oxidation-reduction peak that belongs to the oxidation and reduction of the Ni(II)/Ni(III) couple. This result suggests that the Ni-modified catalytic electrode could be applied as direct methanol fuel-cell catalysts. The catalytic efficiency (in terms of current density) of the Ni-modified catalytic electrodes for methanol oxidation was calculated to be 10.5 mAM^−1^ cm^−2^. we also investigated the stability of the polymeric Ni complex-modified electrode by cyclic voltammetry. The current value of the polymeric Ni complex-modified electrode decreased by only 21.3% from its maximum peak after 120 cycles, which indicates an acceptable stability.

## Discussion

**Figure 8:**
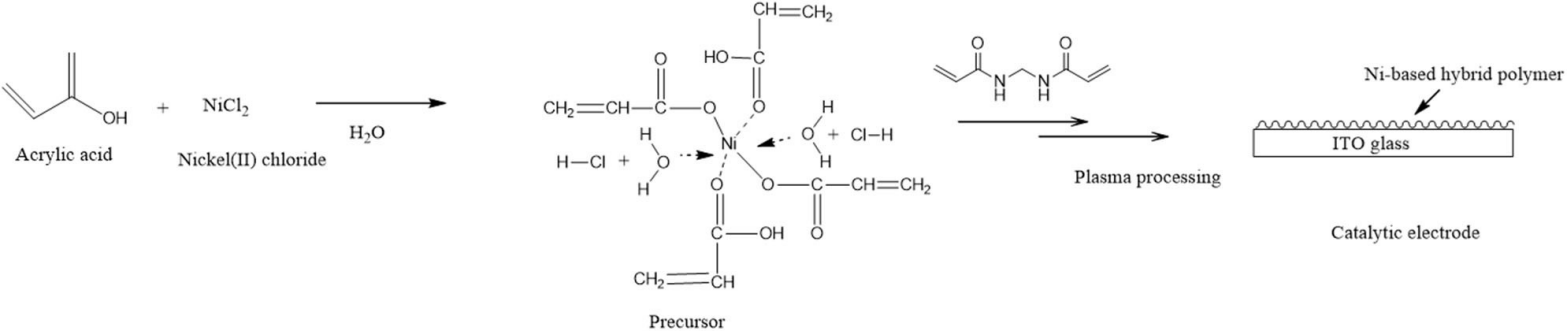
Schematic preparation of the Ni-modified catalytic electrode by cold plasma process for MeOH oxidation.

The inclusion of Ni(II)/Ni(III) redox couples among several polymer metal complexes has received considerable attention in recent years, partly because their behavior in alkaline solutions is reminiscent of hydrogen peroxide species, which are commonly considered as redox mediators between substrates and electrodes in many electro-oxidation processes (Ojani et al., 2009b). In addition, the preparation procedure is usually simple, and the properties of the resulting coating can be carefully controlled. Electropolymerization has been successfully used to deform electrode surfaces with Ni(II) cyclam and similar Ni(II) tetraazamacrocyclic complexes (Ourari et al., 2017) or Ni(II) tetramethyldibezotetraaza(14)anulene (Ciszewski et al., 2003), and the resulting films showed high catalytic activity for electro-oxidation of alcohols and/or other compounds that contain OH and NH_2_. In a previous paper (Bui et al., 2019), polymeric metal complexes with Cu, Fe, and Ni were prepared by an AC plasma process for detecting hydrogen peroxide. This method gives the good challenge which is prepared polymeric metallic complexes with methanol oxidation for methanol fuel-cell catalysts. Therefore, we did the plasma polymerization of the precursor solution as shown in Figure 8. The precursor solution containing acrylic acid, a cross-linking agent, and metal salts can result in complexation between metal ions and acrylic acid via the carboxyl groups.

**Figure 9:**
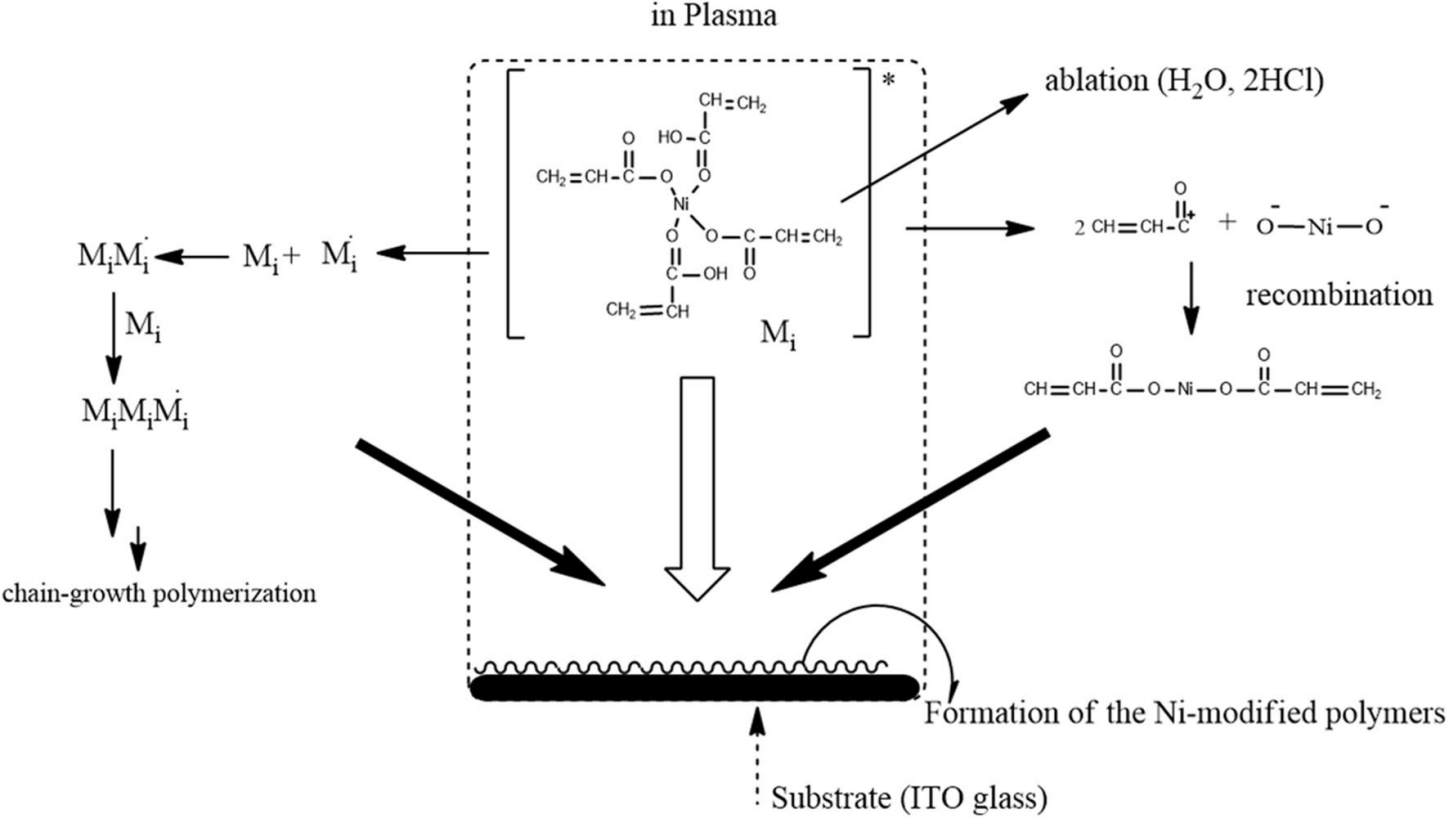
The suggested formation mechanism of the Ni-modified catalyst electrode in cold plasma process.

A plasma is a partially ionized gas composed of radicals, photons, molecules, ions, electrons, and excited species. It is thus a highly reactive mixture, which makes it different from conventional gaseous mixtures. Under the influence of plasma, the resulting catalysts can be very different from those prepared by conventional thermal means. This is the major reason that plasmas have been extensively applied for catalyst preparation (Raoof et al., 2013; Wang et al., 2018). In this experiment, the precursors with metallic complex will be vaporized in a high-temperature environment. Polymeric-Ni complexes with special morphology were controlled through a fast-quenching process using a quench gas, as shown in Figure 9.

**Figure 10:**
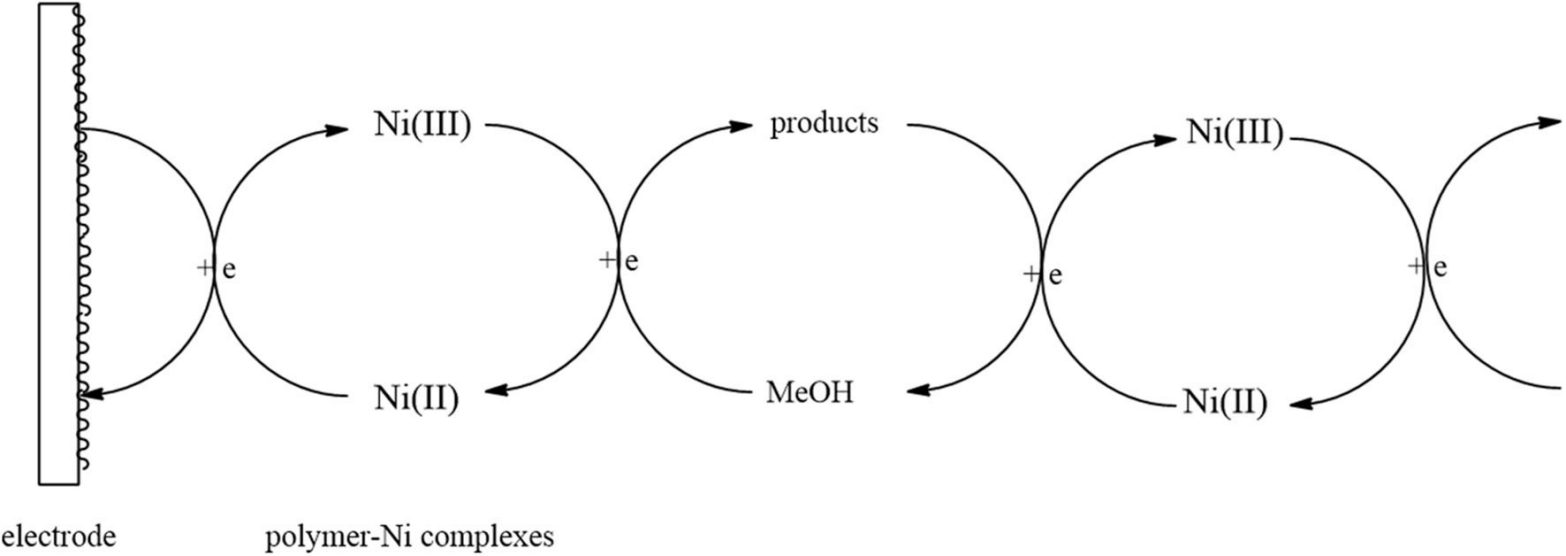
Schematic diagram of the electrocatalytic oxidation of methanol on the surface of a polymer-Ni complex catalytic electrode.

Figure 10 shows the electrocatalytic mechanism of MeOH oxidation on the surface of a Ni-modified ITO electrode. The Ni(II) was first electrochemically oxidized to Ni(III), which then reacted chemically with CH_3_OH and resulted in the CH_3_OH reverted to products such as formic acid or formaldehyde and in the regeneration of the catalyst.

## Conclusions

The Ni-modified (polymeric Ni complex) catalytic electrode for methanol oxidation can be successfully fabricated by one-step cold-plasma deposition of the synthesized precursor solutions. we characterized the fabricated catalytic electrode with polymeric Ni complexes by SEM, contact angle, X-ray photoelectron spectroscopy, cyclic voltammetry, and energy-dispersive X-ray spectroscopy, and evaluated the catalytic efficiency of methanol in PBS solution. From the results, we have drawn the following conclusions.

(1) A Ni-modified catalytic electrode could be fabricated by the easy and simple one-step plasma process without any treatment.
(2) The catalytic activity was calculated at 10.5 mAM^−1^cm^−2^. The Ni-modified catalytic electrode shows higher catalytic activity for methanol oxidation.
(3) The stability of polymeric Ni complex-modified electrodes appears to be acceptable in practical applications.

## Data Availability Statement

The original contributions presented in the study are included in the article/supplementary material, further inquiries can be directed to the corresponding author/s.

## Author Contributions

J-SJ conducted an experiment for this study. I-KY among the results of this study, proved that the Ni-modified catalyst electrode has the electrical catalytic effect of MeOH. WK conducted an analysis on the analysis method and results of the experimental design of this study. S-HC designed the overall synthesis methods and the research plan of this study. All authors contributed to the article and approved the submitted version.

## Conflict of Interest

The authors declare that the research was conducted in the absence of any commercial or financial relationships that could be construed as a potential conflict of interest.
